# The effect of drying temperature on bioactive compounds and antioxidant activity of *Leccinum scabrum* (Bull.) Gray and *Hericium erinaceus* (Bull.) Pers.

**DOI:** 10.1007/s13197-019-04081-1

**Published:** 2019-09-18

**Authors:** Monika Gąsecka, Marek Siwulski, Zuzanna Magdziak, Sylwia Budzyńska, Kinga Stuper-Szablewska, Przemysław Niedzielski, Mirosław Mleczek

**Affiliations:** 1 Department of Chemistry, Poznań University of Life Sciences, Poznan, Poland; 2 Department of Vegetable Crops, Poznań University of Life Sciences, Poznan, Poland; 3 Faculty of Chemistry, Adam Mickiewicz University in Poznań, Poznan, Poland

**Keywords:** Phenolic acids, Organic acids, Antioxidant activity, Ergosterol, Metal content, Mushrooms

## Abstract

In the study the effect of drying temperature on phenolic and organic acid content, total phenolic content, ergosterol content, antioxidant activity and content of 40 elements in fruiting bodies of *Leccinum scabrum* and *Hericium erinaceus* was estimated. The analysis was performed for fresh fruiting bodies and those dried at 20, 40 and 70 °C. Drying resulted in changes in the profile of phenolic and organic acids. Drying generally resulted in losses of the content of total phenolics, ergosterol and antioxidant activity in both species. However, a reduction and an increase of phenolic acids and organic acids were observed. The greatest reduction of the compounds was generally observed at 70 °C. The greatest losses concerned organic acids (some single components and total) (even more than 90% of some compounds). The inhibition of free radicals decreased in the following order: fresh samples > air-dried samples > samples dried at 40 °C > samples dried at 70 °C. The drying temperature affected only selected element contents in fruiting bodies.

**Electronic supplementary material:**

The online version of this article (10.1007/s13197-019-04081-1) contains supplementary material, which is available to authorized users.

## Introduction

*Leccinum scabrum* (Bull.) Gray belonging to the Boletaceae family occurs throughout the northern hemisphere and in Australia and New Zealand. It is a wild growing species forming mycorrhiza with birch. *Leccinum scabrum* is one of the most commonly consumed wild growing mushrooms. The most delicious are the young fruiting bodies and caps. It is suitable for direct consumption, drying or processing. Mushroom shelf life at room temperature after harvest is limited to a few days (Akram and Kwon 2010; Sommer et al. 2010). That is why after the direct harvesting from the natural environment fruiting bodies of *L. scabrum* are available in trade also as a dried product. The conditions after harvest as well as culinary treatments greatly affect the quality of mushrooms (Lin et al. 2017; Roncero-Ramos et al. 2017; Sudheer et al. 2016). *Hericium erinaceus* (Bull.) Pers., also called the lion’s mane mushroom, is known as a medicinal mushroom used in East Asia which demonstrates health-promoting effects including antioxidant, antibacterial, anti-aging, anti-tumor and anti-dementia activity and a neuroprotective effect resulting from the presence of more than 80 kinds of bioactive compounds (Friedman 2015; Li et al. 2016; Lu et al. 2014). It is cultivated in many countries in the world.

Fresh fruiting bodies (especially wild growing) are not available all year round, but consumers can choose dried mushrooms instead. Hot-air drying is a relatively cheap, easily controlled and mostly used drying method (Pendre et al. 2012). However the drying can affect content of some bioactive compounds. It is also important for consumers, who are increasingly appreciating mushrooms not only for their taste but also for their nutritional value and healthy properties associated with the presence of biologically active compounds.

The nutritional and health benefits of mushrooms are connected with polysaccharides, proteins and amino acids (e.g. leucine, lysine, methionine, tryptophan), dietary fibre (mainly in the form of chitin), essential unsaturated fatty acids, as well as macro- and micronutrients and vitamins (B_1_, B_2_, B_12_, C, D, niacin, folic acid), phenolics, organic acids, sterols, alkaloids and terpenoids (Anibal et al. 2015; Heleno et al. 2015; Jedidi et al. 2017; Reid et al. 2017; Sułkowska-Ziaja et al. 2012).

Phenolic compounds are a major group of non-nutrient bioactive substances with antioxidant, antibacterial and anti-inflammatory properties which may promote natural defense mechanisms in humans (Chen et al. 2017b; Heleno et al. 2015; Moro et al. 2012; Nowacka et al. 2014; Souilem et al. 2017). Phenolic compounds may modify metabolic and physiological functions of the human organism and exhibit health-promoting effects (Chen et al. 2017b; Moro et al. 2012; Souilem et al. 2017).

Ergosterol (previtamin D_2_) is a sterol contained in cell membranes which has beneficial effect on human health and many physiological functions due to its antibacterial, anti-inflammatory and anticancer properties, the potential to reduce the incidence of cardiovascular disease as well as inhibition of cyclooxygenase (COX) activity (Chen et al. 2017b; Hu et al. 2006; Phillips et al. 2011; Shao et al. 2010; Zhang et al. 2002). Ergosterol under the influence of radiation is converted to vitamin D_2_ (a form of vitamin D), essential for human metabolism and found naturally in fungi and plants (Mattila et al. 2002; Phillips et al. 2011; Villares et al. 2014).

Organic acids may prevent various diseases because of their antioxidant properties (citric, malic and succinic acids), antibacterial properties (oxalic acid) and anti-inflammatory properties (formic acid) (Altmeyer et al. 1994; Baati et al. 2011; Kwak et al. 2016; Valentão et al. 2005). The metabolites also prevent browning of mushrooms, extend the shelf life and are also responsible for the taste and aroma of mushrooms (Barros et al. 2013; Brennan et al. 2000; Valentão et al. 2005).

The antioxidant properties of bioactive molecules play an important role in preventing damage caused by free radicals (e.g. age-related disorders and cancer) (Genkinger et al. 2004; Shukla and Singh 2007).

In the study the effect of drying temperature on the content of phenolic and organic acids and antioxidant activity in two different species of mushroom was estimated. Additionally the influence of drying temperature on the content of 40 elements was also estimated. The newly presented data provide some important nutritional information about the differences in fresh and dried mushrooms’ chemical composition.

## Materials and methods

### Mushroom samples

Wild *L. scabrum* sample*s* were collected from Wielkopolska Region in Poland (52°40′N 17°10′E) in mid September 2017.

*Hericium erinaceus* was cultivated on a mixture of beech, birch and oak sawdust (3:1:1 vol) supplemented with wheat bran in the amount 25%, corn flour (1%), sucrose (1%) and CaSO_4_ (1%) in relation to the substrate dry matter. The mixture was watered to the moisture content of 60%, placed in polypropylene bags (1 kg), closed with a cotton cork and then sterilized (121 °C, 1.5 h). After cooling to room temperature the substrate was inoculated with grain mycelium (20 g per bag) and incubated (25 ± 1 °C and 80–85% air humidity).The cotton cork was removed after the substrate became overgrown with mycelium. Then the bags were placed in the cultivation room (16–18 °C and air humidity 85–90%) and lighted with a fluorescent lamp (day-light) for 12 h per day (200 lx intensity of irradiation). The fruiting bodies were harvested successively when they reached maturity. The fruiting bodies of both species were divided into 4 analysis groups as follows: fresh fruiting bodies, dried at air temperature (20 °C, 48 h), dried at 40 °C (12 h) and 70 °C (7 h) in an oven.

### Extraction

The extraction of phenolic and organic acids was carried out on fresh and dried samples. The fresh samples of both species were homogenized, while the dried samples were ground to a fine powder. The samples were mixed with 80% methanol and shaken for 12 h at room temperature for extraction of phenolic acids. Organic acids were extracted by water in an ultrasound bath at ambient temperature. Then the samples were centrifuged at 3000 rpm, evaporated to dryness at 40 °C with rotary vacuum and stored at − 20 °C before analyses. For analysis the extracts were redissolved in 1 mL of deionized water (for organic acids) and in 80% methanol (phenolics).

### Total phenolic content

The content of total phenolics (TP) was determined using the Folin–Ciocalteu assay according to Singleton and Rossi (1965) with some modification. The mixture of the extract (100 µL) and the Folin–Ciocalteu reagent (1 mL diluted with distilled H_2_0; 1:1, v/v) and Na_2_CO_3_ (3 mL, 20%) was incubated for 30 min in darkness at room temperature. Next the absorbance was measured at 765 nm. The results were expressed as mg of gallic acid equivalent (GAE) per g of dry weight (DW).

### UPLC analysis of phenolic compounds

Phenolic acids and organic acids were identified on an ACQUITY UPLC H-Class System and PDA eλ Detector (Waters Corporation, Milford, MA, USA). For separation of the compounds an Acquity UPLC BEH C_18_ column (2.1 mm × 150 mm, 1.7 µm, Waters) thermostated at 35 °C was used. The gradient elution was performed with water and acetonitrile (both containing 0.1% formic acid, pH = 2) at a flow rate of 0.4 mL/min. Compounds were identified by comparing retention time of the analyzed peaks with the retention time of standards or by adding a specific amount of the standard to the analyzed samples and a repeated analysis. Detection was carried out in a Waters Photodiode Array Detector at λ = 280 nm and λ = 320 nm using an external standard.

### Evaluation of DPPH radical scavenging activity

The scavenging effect against the DPPH˙ radical was carried out according to Dong et al. (2012). 1 mL of extract at concentration of 10 mg/mL was mixed with 2.7 mL of methanolic solution of 2,2-diphenyl-1-picrylhydrazyl (DPPH) radicals at concentration of 6 µmol/L. The mixture was incubated in the dark for 60 min. Then the absorbance of the mixture was measured at 517 nm. The ability to scavenge DPPH radicals was expressed as follow:

$$ {\text{DPPH}}\;{\text{inhibition}}\;\left( \% \right) = [({\text{A}}_{\text{control}} - {\text{A}}_{\text{sample}} )/{\text{A}}_{\text{control}} )] \times 100 $$

### Evaluation of ABTS radical scavenging activity

The scavenging effect against the ABTS radical was carried out as described earlier (Gąsecka et al. 2015). A mixture of 7 mol/L ABTS (2,2′-azino-bis-(3-ethylbenzothiazoline-6-sulfonic acid) diammonium salt) and 2.45 mol/L potassium persulfate solution was kept in darkness overnight to generate radicals. Then 100 µL of methanolic extract was mixed with the ABTS solution. After 10 min, the absorbance was measured at 734 nm. The radical scavenging activity was calculated according to the formula above.

### Extraction and determination of ergosterol

The extraction procedure was according to Perkowski et al. (2008) with some modification. The mixture of mushroom samples, pure methanol and 2 M NaOH were irradiated twice in a microwave oven for 15 s, cooled, and mixed with 1 M HCl. Then the mixture was extracted with pentane three times. Analyses were carried out in an ACQUITY UPLC H-Class System coupled with a PDA eλ Detector (Waters Corporation, Milford, MA, USA) using an ACQUITY UPLC HSS T3 C_18_ column (150 mm × 2.1 mm, particle size 1.8 µm) (Waters, Ireland) and 1.7 m ACQUITY UPLC BEH C18 VanGuard Pre-column (Waters, Ireland). The mobile phase was a mixture of methanol, acetonitrile and water (v:v:v; 85:10:5). The isocratic elution with the flow rate of 0.5 mL/min was applied.

### Preparation of samples for analysis of element contents

Both dried and fresh samples were digested with 7 mL of concentrated nitric acid (65% nitric acid, Merck, Darmstadt, Germany) in closed Teflon containers in the microwave digestion system Mars 6 Xpress (CEM, Matthews, USA). Samples were then filtered through paper filters and diluted with water to a final volume of 15.0 mL. Each of the samples was processed in 3 replicates.

### Instruments and quality control

The inductively coupled plasma optical emission spectrometer Agilent 5110 ICP-OES (Agilent, USA) was used for determination of 40 elements (Al, Ba, Be, Ca, Cd, Ce, Cr, Cu, Dy, Er, Eu, Fe, Gd, Hg, Ho, Ir, K, La, Lu, Mg, Mn, Na, Nd, Ni, P, Pb, Pr, Rb, Sb, Sc, Se, Sm, Sn, Sr, Tb, Tl, Tm, Y, Yb, Zn) under common conditions (see Online Resource). ICP commercial analytical standards—mixed mono- and multi-elemental (Romil, England)—were used for the calibration. The selected wavelengths and validation parameters were as follows: the detection limits obtained follow 3-sigma criteria at the level of 0.0X mg/kg dry weight (DW); precision and upper range of calibration curves are presented in Table 2. Uncertainty for the complete analytical process (including sample preparation) was at the level of 20%. Traceability was checked using the standard reference materials CRM S-1 = loess soil; CRM NCSDC (73,349) = bush branches and leaves; CRM 2709 = soil; CRM 405 = estuarine sediments; and CRM 667 = estuarine sediments with the recovery acceptable for all the elements (80–120%).

### Statistical analysis

The results in tables and in figures were expressed as the mean value of three replicates (n = 3) and the standard deviation. Differences between mean values were evaluated by one-way analysis of variance (ANOVA) followed by post hoc Tukey’s test. Statistical analysis was performed using STATISTICA 13.1.

## Results and discussion

### Total phenolic content

**Fig. 1:**
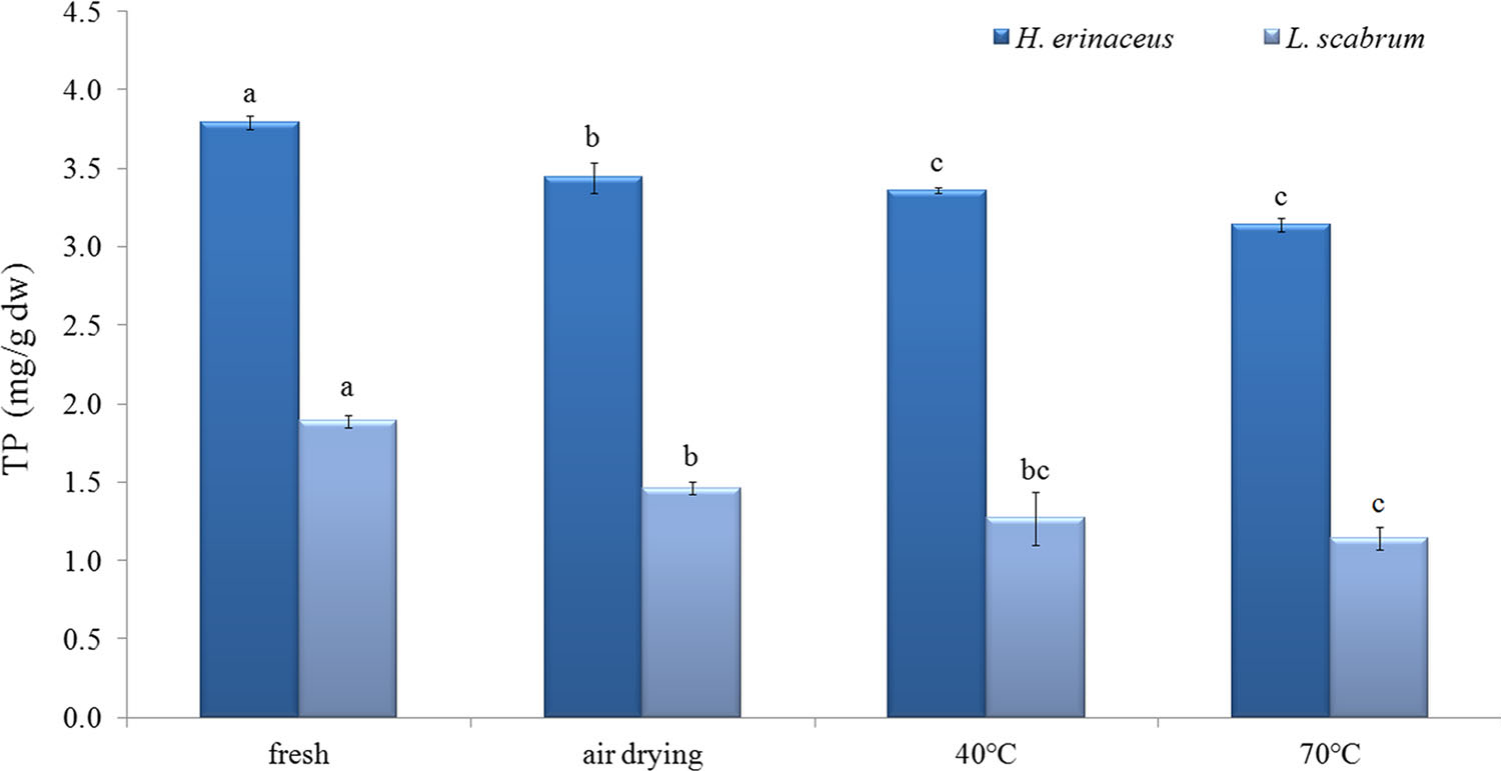
Impact of drying temperature on total phenolic content

Total phenolic content was nearly two-fold higher in *H. erinaceus* than in *L. scabrum* (Fig. 1). TP content in *H. erinaceus* was lower than in the study presented by Yildiz et al. (2015). The drying method significantly affected TP content in both species. The decrease was confirmed for each drying temperature in comparison to fresh samples. For *H. erinaceus* it was a 17% reduction of TP (from 3.79 to 3.14 mg/g DW), while for *L. scabrum* it was a 40% reduction of TP (from 1.89 to 1.14 mg/g DW) at 70 °C in comparison to fresh. The results were in agreement with some studies on mushrooms, fruits and seeds, which contradict the reduction of TP under high-temperature drying (Juhaimi et al. 2018; Sim et al. 2017). The loss of TP (up to 25%) during drying at high-temperature was also confirmed for edible mushroom *Phlebopus colossus* (Liaotrakoon and Liaotrakoon 2018).

Phenolics are molecules sensitive to temperature, and the changes of the content (both reduction and increase) with different drying temperature was indicated (Jaworska et al. 2014; Jiang et al. 2017; Sim et al. 2017; Yang et al. 2017). The oxygen atmosphere was responsible for their oxidation (Hamrouni-Sellami et al. 2012). In *Grifola frondosa* mycelia the drying in an oven at 70 °C reduced the TP content only from 17.0 to 16.6 mg GAE/g (Sim et al. 2017). In *Lentinula edodes* an increase of TP was observed under hot air drying (Yang et al. 2017), because the phenolic compounds are substrates of lignin, whose more intense accumulation was observed as a result of high temperature drying. The reduction of phenolic compounds during drying may be the result of the activation of oxidative enzymes (polyphenol oxidase and peroxidase) (An et al. 2016) and the result of changes in the structure of the phenolic compounds (Toor and Savage 2006). Sezer et al. (2017) did not find any relation between TP and drying temperate of mushrooms. Oven drying at 43 °C did not affect TP or total flavonoid content in *Pleurotus ostreatus* (Mutukwa et al. 2019). On the other hand, drying at air temperature for 7 days significantly increased TP (from 8.77 to 119.8 mg GAE/g) in the mushroom *Amanita zambiana* due to the better extractability of bound polyphenols as a result of cell wall destruction after drying (Reid et al. 2017). Jaworska et al. (2014) also found that TP was elevated; however, total flavonoids were lost in *Boletus edulis* during drying. Šumić et al. (2017) suggested that increase of temperature significantly decreased TP in *Cantharellus cibarius*, but they predicted that simultaneous use of temperature of 55 °C and vacuum pressure (10 kPa) would preserve phenolic compounds and cause their increase. A study on the effect of drying temperature on TP in jujube fruits showed that phenolics were rather stable at 55 °C and only a small reduction of TP content was observed, but the drying and storage at ambient temperature elevated TP (Pu et al. 2018). In citrus seeds the drying process significantly affected TP content (Juhaimi et al. 2018). The temperatures of 60 and 70 °C elevated or reduced the TP content depending on the citrus seeds, but for all analyzed samples a significant drop was confirmed at 80 °C (Juhaimi et al. 2018).

In the profile of *H. erinaceus*, caffeic, chlorogenic, ferulic, gallic, 4-hydroxybenzoic, salicylic, sinapic, syringic, *t*-cinnamic and vanillic acid were quantified (Table 1). Gallic acid was the prevalent phenolic acid in the profile of *H. erinaceus* (21.5 µg/g DW). In comparison to our earlier study some phenolic compounds were not detected (Gąsecka et al. 2016). To our knowledge, the composition of the phenolic compounds of *H. erinaceus* has been studied in only a few works. Gallic, ferulic, syringic and vanillic acids were quantified in another study as well (Yildiz et al. 2015), while 4-hydroxybenzoic, ferulic and syringic acids were detected by Li et al. (2012). The drying temperature resulted in the reduction of the content of the acids (from 2 to 37-fold). However, the content of 4-hydroxybenzoic and sinapic acid increased, achieving the highest value at 70 °C. No significant changes in the content of caffeic, ferulic, salicylic, syringic and *t*-cinnamic acids between different drying temperatures were observed. In the profile of phenolic acids of *L. scabrum t*-cinnamic and protocatechuic acids were dominant. Additionally, fruiting bodies were characterized by presence of caffeic, chlorogenic, 2,5-dihydroxybenzoic, ferulic, 4-hydroxybenzoic, salicylic, syringic and vanillic acids. Nowacka et al. (2014) also found caffeic, ferulic, 4-hydroxybenzoic and protocatechuic acids in fruiting bodies of *L. scabrum*. The drying did not affect salicylic acids, but contents of other acids were lower in dried samples. There were no significant changes in the content of ferulic, syringic and vanillic acids between different temperatures of drying. Only air drying had no impact on caffeic, chlorogenic, 2,5-dihydroxybenzoic, 4-hydroxybenzoic and syringic acids. For other acids drying reduced the content. For both species a significant drop of total phenolic acids (sum) was confirmed in drying samples in comparison to fresh samples. Knowledge of changes in the phenolic profile of mushrooms under the drying is very scarce. However, the results obtained for various fruits and vegetables indicated the effect of the drying temperature on the content of phenolic acids. In black rice ferulic, caffeic, *p*-coumaric and gallic acids were found to be thermally unstable (Lang et al. 2019). Other changes were observed in jujube fruits, because the content of chlorogenic, *p*-hydroxybenzoic, caffeic, *p*-coumaric and ferulic acids in dried jujube fruits was significantly higher than in fresh products (Pu et al. 2018). The contents of phenolic compounds of citrus seeds were modified by drying temperate (Juhaimi et al. 2018). Contents of gallic and 3,4 dihydroxybenzoic acids increased at 60 and 70 °C, while the content decreased at 80 °C. The content of caffeic, *p*-coumaric, trans-ferulic, and syringic acids changed during drying, but both elevation and reduction of the content were observed (Juhaimi et al. 2018). The loss of phenolic compounds may be a result of activation of oxidative enzymes such as polyphenol oxidase and peroxidase (An et al. 2016), and changes in chemical structure of phenolic compounds via binding them to proteins (Toor and Savage 2006). Additionally, in mushrooms during drying, morphological changes may occur in fruiting bodies associated with lignification that affect the phenolic compounds, which are substrates of the lignin (Yang et al. 2017).

**Table 1:** Phenolic acid content (µg/g DW)in fruiting bodies of *H. erinaceus* and *L. scabrum* at different temperature of drying

a	Caffeic	Chlorogenic	2,5-Dihydroxybenzoic	Ferulic	Galic	4-Hydroxubenzoic	Protocatechiuc
*H. erinaceus*							
Fresh	4.430^a^ ± 0.153	2.688^a^ ± 0.147	nd	6.254^a^ ± 0.146	21.508^a^ ± 0.615	5.538^c^ ± 0.263	nd
Air drying	0.120^b^ ± 0.029	1.104^c^ ± 0.129	nd	1.246^b^ ± 0.098	11.365^d^ ± 0.459	5.439^c^ ± 0.210	nd
40 °C	0.124^b^ ± 0.011	1.275^bc^ ± 0.177	nd	1.436^b^ ± 0.095	13.872^c^ ± 0.199	6.267^b^ ± 0.166	nd
70 °C	0.142^b^ ± 0.006	1.444^b^ ± 0.112	nd	1.571^b^ ± 0.234	15.445^b^ ± 0.520	6.961^a^ ± 0.158	nd
*L. scabrum*							
Fresh	0.333^a^ ± 0.210	0.228^a^ ± 0.014	0.625^a^ ± 0.034	0.185^a^ ± 0.016	nd	0.68^a^ ± 0.034	5.039^a^ ± 0.124
Air drying	0.324^ab^ ± 0.003	0.233^a^ ± 0.009	0.576^ab^ ± 0.008	0.148^b^ ± 0.012	nd	0.708^a^ ± 0.008	4.650^b^ ± 0.116
40 °C	0.293^bc^ ± 0.130	0.214^a^ ± 0.013	0.541^bc^ ± 0.017	0.129^b^ ± 0.006	nd	0.657^ab^ ± 0.014	4.185^c^ ± 0.061
70 °C	0.284^c^ ± 0.007	0.182^b^ ± 0.006	0.500^c^ ± 0.008	0.123^b^ ± 0.002	nd	0.623^b^ ± 0.009	4.146^c^ ± 0.061

	Salicylic	Sinapic	Syringic	*t*-Cynnamic	Vanilc	Sum
*H. erinaceus*						
Fresh	5.286^a^ ± 0.229	0.583^d^ ± 0.035	1.620^a^ ± 0.096	1.189^a^ ± 0.011	5.308^a^ ± 0.183	64.763^a^ ± 1.066
Air drying	1.047^b^ ± 0.072	2.152^c^ ± 0.149	1.137^b^ ± 0.116	0.229^b^ ± 0.010	2.531^d^ ± 0.156	26.371^c^ ± 0.858
40 °C	1.141^b^ ± 0.149	2.554^b^ ± 0.283	1.246^b^ ± 0.105	0.272^b^ ± 0.145	2.976^c^ ± 0.097	31.163^b^ ± 0.184
70 °C	1.363^b^ ± 0.080	2.948^a^ ± 0.168	1.359^b^ ± 0.076	0.302^b^ ± 0.015	3.264^b^ ± 0.128	34.800^b^ ± 1.014
*L. scabrum*						
Fresh	0.219^a^ ± 0.013	nd	0.558^a^ ± 0.023	13.255^a^ ± 0.267	1.166^a^ ± 0.060	22.289^a^ ± 0.539
Air drying	0.219^a^ ± 0.008	nd	0.535^ab^ ± 0.010	11.165^b^ ± 0.746	0.978^b^ ± 0.010	19.536^b^ ± 0.712
40 °C	0.227^a^ ± 0.014	nd	0.531^ab^ ± 0.010	9.684^c^ ± 0.267	0.964^b^ ± 0.009	17.424^c^ ± 0.279
70 °C	0.220^a^ ± 0.011	nd	0.506^b^ ± 0.012	9.134^a^ ± 0.106	0.942^b^ ± 0.011	16.659^c^ ± 0.183

Mean values (n = 3) and standard deviations; values within columns for each species of mushroom separately followed by the different letters are significantly different (*P* < 0.05) according to Tukey’s HSD test (ANOVA)

### Antioxidant activity

**Fig. 2:**
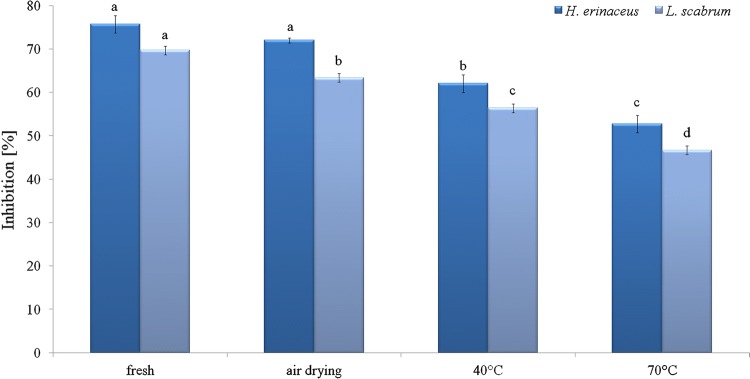
DPPH radical scavenging activity (RSA, %) of fresh and dried samples

**Fig. 3:**
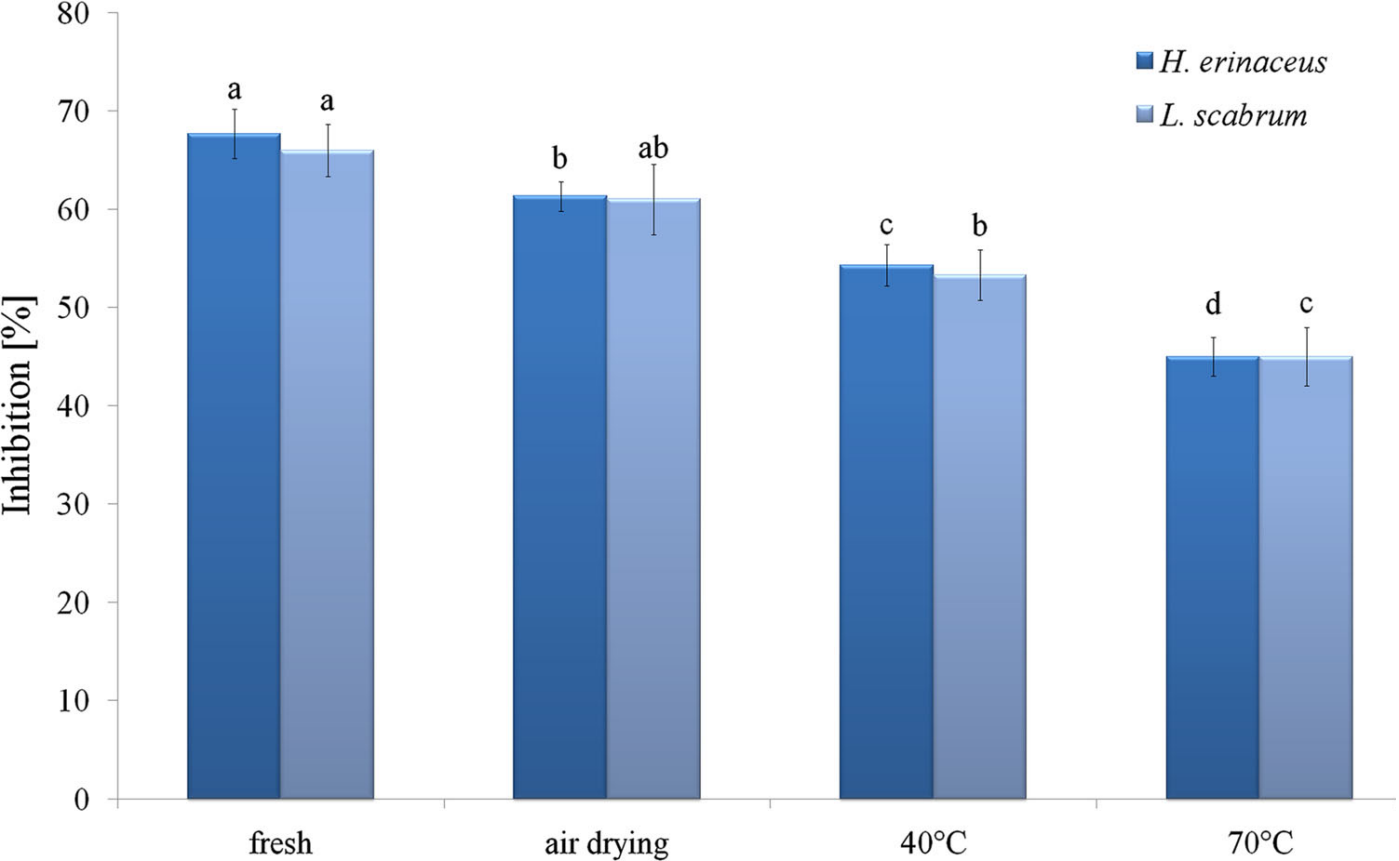
ABTS radical scavenging activity (RSA, %) of fresh and dried samples

The ability of *H. erinaceus* and *L. scabrum* to scavenge DPPH and ABTS radicals is shown in Figs. 2 and 3. Higher potential to scavenge free radicals was exhibited by *H. erinaceus.* The drying resulted in reduction of radical scavenging ability. The scavenging activity for both species was in the order: fresh samples > air-dried samples > samples dried at 40 °C > samples dried at 70 °C. The inhibition of DPPH radicals decreased from 76 to 53% for *H. erinaceus* and from 70 to 47% for *L. scabrum* after drying (mainly from 40 °C in comparison to fresh samples). The inhibition of ABTS radicals decreased from 68 to 45% for *H. erinaceus* and from 66 to 45% for *L. scabrum.* The results were in agreement with those of Liaotrakoon and Liaotrakoon (2018), who demonstrated a drastic reduction in the ability to scavenge DPPH radicals during drying of *P. colossus.* The drying in an oven also significantly reduced DPPH radical scavenging activity of *G. frondosa* in comparison to fresh mushroom (Sim et al. 2017). Free radical scavenging activity expressed increased from 27.23 to 84.06% for *P. ostreatus* and from 33.22 to 76.46% for *Agaricus bisporus* in non-dried samples and those dried at 80 °C (Reid et al. 2017). An increase of antioxidant activity during drying was also observed for *B. edulis* (Jaworska et al. 2014). The air-dried samples of okra showed lower antioxidant activity in comparison to fresh samples and TP was highly correlated with DPPH-scavenging ability (Juhaimi et al. 2018). However, in citrus seeds the drying process at 60 and 70 °C enhanced antioxidant activity while at 80 °C a decrease was observed (Juhaimi et al. 2018). The ability to scavenge free radicals is connected with phenolic compounds (Šumić et al. 2017). Additionally, Sim et al. (2017) suggested that heating of the mycelium interrupts its ability to donate hydrogen.

### Organic acids

Identification of the quantitative analysis of the main organic acids found in fruits, vegetables and mushrooms is considered very important for the assessment of their quality. These acids affect not only the taste, but also their stability, nutrition, acceptability and maintaining quality (Igual et al. 2012; Gao et al. 2012, Colina-Coca et al. 2014, Barros et al. 2013). The beneficial health effects and bioactive properties of some organic acids (e.g. tartaric, malic, citric or succinic acids) are well-known and include antioxidant and antimicrobial, and acidifying properties (Barros et al. 2013; Valentão et al. 2005; Stojković et al. 2017). Metabolites play an important role in mushroom flavour (Ribeiro et al. 2006). Organic acids are more stable during processing and storage than other components such as pigments and flavour compounds (Fernandes et al. 2014). However, information with respect to how their content is affected by various drying methods was, to our knowledge, not available.

The major content of organic acids was observed for fresh mushroom samples, in both studied species (Table 2). Quinic and acetic acids were present in the largest amount in *H. erinaceus* samples (119.22 and 88.18 µg/g DW), representing 55.78 and 41.26%, respectively, of the total organic acid content. In *L. scabrum* samples oxalic, succinic and citric acids were dominant (148.19, 34.10 and 28.37 µg/g DW), and accounted for 70.34, 16.19 and 13.47%, respectively. The drying process under different temperatures significantly affected the content of organic acids compared to fresh mushroom samples (Table 2). The content of total analysed organic acids in dried samples of *H. erinaceus* was ~ 2.4 to 6.9-fold lower (from 213.73 (fresh) to 73.74 and 71.62 for air and 40 °C drying, respectively, while under 70 °C this fall was to 36.96 µg/g DW). In the case of *L. scabrum* this decrease was definitely higher than that observed in *H. erinaceus* and ranged from ~ 5.8 to ~ 35-fold (from 210.66 to 31.44, 6.12 and 4.74 µg/g DW under air, 40 °C and 70 °C drying process method, respectively). What should be noted is, that significant decreases were particularly observed for dominant acids obtained in mushroom species samples, even above 95% (e.g. content of quinic acid in *H. erinaceus* decreased from 119.22 in fresh samples to 0.03 µg/g DW in samples dried under 70 °C and oxalic acid in *L. scabrum* where the decrease ranged from 148.19 in fresh samples to 0.86 µg/g DW in samples dried under 70 °C. As can be seen, the drying treatments led to a significant decrease (*p* < 0.05) in the content of major acids and their total sum, which is also connected with the processing method (higher temperature lower content of dominant acid). In *H. erinaceus* samples, organic acids such as malic, citric, succinic and lactic showed significant differences, associated with their content increase. The content of malic acid increased more than twofold (from 1.39 to 3.33 and 2.87 under air and 40 °C process drying, respectively). The increase in the amount of malic acid extracted from samples treated in the medium dryness process may be caused by activation of its metabolic formation during the glycolytic pathway as a product of the transformation of succinic acid, obtained data for mushrooms correspond with the results described in the literature for other vegetables and fruits (Colina-Coca et al. 2014; Gao et al. 2012). This could be an effect similar to that described by Slatnar et al. (2011). In dried samples of fig fruits, the organic acids were more concentrated, because of lower water content (Slatnar et al. 2011). However, for *L. scabrum* only a decrease in the content of the studied acids was observed as a consequence of drying under higher temperature, which is likely to be connected with decomposition and degradation of the studied acids (Priecina et al. 2018).

**Table 2:** Organic acids content (µg/g DW) in fruiting bodies of *H. erinaceus* and *L. scabrum* at different temperature of drying

	Acetic	Citic	Fumaric	Lactic	Malic	Oxalic	Quinic	Succinic	Sum
*H. erinaceus*									
Fresh	88.185^a^ ± 2.481	4.016^c^ ± 0.114	nd	0.924^c^ ± 0.026	1.386^c^ ± 0.040	nd	119.220^a^ ± 3.355	nd	213.731^a^ ± 6.014
Air drying	25.888^b^ ± 0.511	6.681^b^ ± 0.511	0.248^a^ ± 0.004	3.140^b^ ± 0.062	3.326^a^ ± 0.066	0.048^b^ ± 0.001	15.836^b^ ± 0.313	18.572^a^ ± 0.366	73.739^b^ ± 1.456
40 °C	1.829^d^ ± 0.051	54.412^a^ ± 1.531	nd	11.126^a^ ± 0.313	2.867^b^ ± 0.081	0.041^b^ ± 0.001	0.470^c^ ± 0.008	0.879^c^ ± 0.025	71.624^b^ ± 2.016
70 °C	12.702^c^ ± 0.204	7.904^b^ ± 0.127	nd	nd	nd	0.161^a^ ± 0.004	0.031^c^ ± 0.001	16.166^b^ ± 00.260	36.964^c^ ± 2.595
*L. scabrum*									
Fresh	nd	28.372^a^ ± 1.117	nd	nd	nd	148.187^a^ ± 4.942	nd	34.099^a^ ± 1.320	210.658^a^ ± 4.214
Air drying	nd	0.391^b^ ± 0.008	nd	28.637^a^ ± 1.514	nd	1.981^b^ ± 0.041	nd	0.436^b^ ± 0.013	31.445^b^ ± 2.540
40 °C	nd	0.359^b^ ± 0.008	nd	3.801^b^ ± 0.093	nd	1.835^b^ ± 0.070	nd	0.422^b^ ± 0.016	6.417^c^ ± 0.024
70 °C	nd	0.274^b^ ± 0.012	nd	3.600^b^ ± 0.158	nd	0.863^b^ ± 0.045	nd	nd	4.737^c^ ± 0.211

Mean values (n = 3) and standard deviations; values within columns for each species of mushroom separately followed by the different letters are significantly different (*P* < 0.05) according to Tukey’s HSD test (ANOVA)

### Ergosterol

**Fig. 4:**
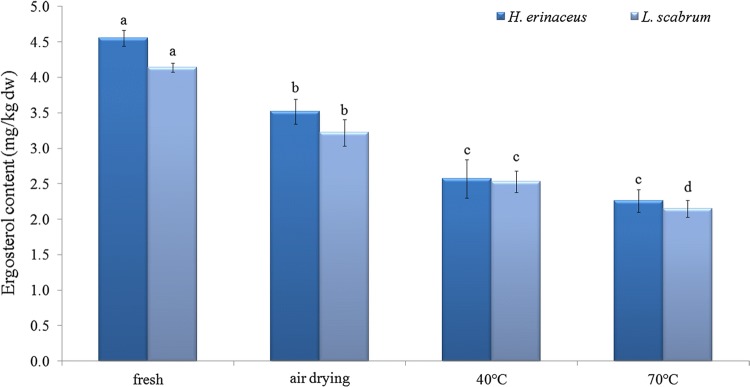
Ergosterol content in fresh and dried samples

Ergosterol was detected in all samples, with the highest level in fresh samples (Fig. 4). Differences in ergosterol content were found between fresh and dried samples. All methods of drying reduced the content of the sterol. The strongest effect was confirmed at 70 °C for *H. erinaceus* and *L. scabrum*. The drop was about 50 and 48% respectively. Ergosterol is the principal sterol in mushrooms with content related to the species. Losses of the content under drying were detected in some studies (Bernaś 2017; Sławińska et al. 2016), while drying in low temperature indicated that ergosterol content was lower in 15 °C than 5 °C and 10 °C (Vallespir et al. 2019). The studies on *A. bisporus* confirmed about 30% loss of ergosterol content in air-dried mushrooms in comparison to fresh samples (Bernaś 2017). Additionally, the study revealed that drying method and temperature of storage after drying significantly affected the reduction of the ergosterol level. A decrease of ergosterol content was also found in dried samples of *A. bisporus* and other species of mushroom—*P. ostreatus* and *L. edodes* (Sławińska et al. 2016). Moreover, in the case of hot air dried mushrooms their ability to produce vitamin D after drying under UVB irradiation was confirmed. Storage temperature and UV radiation influence the stability of the ergosterol extract—higher oxidation and production of ergosterol peroxides occurs with increasing temperature, while UV radiation promotes transformation into vitamin D (Villares et al. 2014). Thus the losses of ergosterol in samples of *L. scabrum* and *H. erinaceus* were the effect of drying method, the time of storage and access to light.

### Element contents

In selected mushrooms the analysis of 40 elements was performed. The obtained results were divided into the following groups of elements: minerals, trace elements, heavy rare earth elements (HREEs) and light rare earth elements (LREEs) (Table 3). The results obtained for minerals (Ca, K, Mg, Na and P) show that the content of elements in fresh fruiting bodies was dependent on the species. The mushroom species richest in Ca, Mg, Na and P was *H. erinaceus*, while *L. scabrum* contained a higher content of K. The temperature used during the drying reduced the content of Ca, Mg and P in *H. erinaceus*, while in *L. scabrum* Ca, K, Mg, Na and P content decreased after drying. It is difficult to explain why the content of selected elements decreased with an increase in drying temperature. One possible solution could be varied durability of metal complexes with metallothioneins (MTs) being a ubiquitous class of low molecular weight proteins (Muenger and Lerch 1985). Unfortunately, according to our present studies, such an explanation can be real for copper-MTs complexes only. Such complexes can be relatively easy to destroy because of the low durability of numerous MT in higher temperature what confirm analytical studies using a new method for MTs determination (Ryvolova et al. 2011). The results obtained for trace elements (Al, Ba, Be, Cd, Cr, Cu, Fe, Hg, Ir, Mn, Ni, Pb, Rb, Sb, Se, Sn, Sr, Tl, Zn) show that higher contents of them generally are accumulated in fresh fruiting bodies for *H. erinaceus.* The temperature of drying significantly modified the selected trace element contents similarly to the observation of Garba and Oviosa (2019), who dried *Vernonia amygdalina* using air, sun, oven and solar drying. The use of different drying methods (different temperatures) caused changes in the content of selected elements (minerals, Fe, Pb, Cu). In spite of slight changes, the decrease in content of these elements was significant (α = 0.05) for Ba, Cd, Cr, Hg, Ir, Mn, Ni, Rb, Se, Sn and Zn. The obtained results show the highest content of Al, Ba, Cu, Hg, Pb, Se and Tl in *L. scabrum* fresh fruiting bodies and that the increase of temperature of drying was associated with a significant decrease in content of these toxic elements.

**Table 3:** Content of elements (mg/kg DW) in *H. erinaceus* and *L. scabrum* exposed to different temperatures of drying process

Element	*H. erinaceus*				*L. scabrum*			
	Fresh weight	Temperature of drying (°C)			Fresh weight	Temperature of drying (°C)		
		Air drying	40	70		Air drying	40	70
*Minerals*								
Ca	406.66^a^	387.14^b^	361.43^c^	329.39^d^	296.67^a^	285.33^b^	293.33^ab^	180.83^d^
K	35,645^a^	23,710^a^	31,472^a^	42,700^a^	37,796^a^	37,874^a^	32,677^b^	29,858^c^
Mg	922.00^a^	907.26^ab^	879.33^b^	846.92^c^	807.93^b^	875.96^a^	844.46^a^	789.43^b^
Na	246.91^a^	236.94^bc^	240.07^ab^	229.27^c^	221.53^a^	215.48^ab^	211.52^b^	208.58^b^
P	6600^a^	6374^c^	6209^c^	6457^b^	6365^b^	6473^a^	6498^a^	6035^c^
*Trace elements*								
Al	3.52^c^	3.86^a^	3.82^a^	3.65^b^	2.41^a^	2.23^b^	2.18^c^	1.60^d^
Ba	1.90^a^	1.92^a^	1.81^b^	1.60^c^	0.87^a^	0.81^b^	0.80^c^	0.69^d^
Be	< 0.01	< 0.01	< 0.01	< 0.01	0.10^a^	0.09^a^	0.10^a^	0.09^a^
Cd	1.53^a^	1.32^b^	1.31^b^	1.08^c^	0.55^b^	0.53^c^	0.55^b^	0.64^a^
Cr	3.25^a^	3.21^b^	3.07^c^	2.73^d^	3.55^b^	3.18^c^	3.08^d^	4.29^a^
Cu	32.9^b^	33.3^a^	32.8^b^	33.3^a^	19.6^a^	18.1^c^	19.5^b^	17.0^d^
Fe	19.14^a^	19.16^a^	16.93^a^	17.85^a^	15.74^a^	15.96^a^	14.05^b^	16.01^a^
Hg	0.49^a^	0.49^a^	0.47^ab^	0.30^b^	0.45^a^	0.41^b^	0.26^d^	0.31^c^
Ir	0.16^a^	0.14^b^	0.13^c^	0.14^b^	0.17^b^	0.18^a^	0.16^c^	0.18^a^
Mn	17.24^a^	14.32^b^	15.32^ab^	14.17^b^	17.23^a^	17.35^a^	17.78^a^	17.24^a^
Ni	2.08^a^	2.06^b^	1.98^d^	2.01^c^	1.35^a^	1.28^a^	1.36^a^	1.62^a^
Pb	1.10^a^	0.93^ab^	0.91^ab^	0.85^b^	1.42^a^	1.32^c^	1.37^b^	1.14^d^
Rb	36.01^a^	34.02^c^	35.12^b^	30.29^d^	35.94^b^	35.55^c^	36.41^a^	32.91^d^
Sb	0.85^b^	0.86^a^	0.81^c^	0.43^d^	0.71^b^	0.69^c^	0.62^d^	0.90^a^
Se	0.97^a^	0.87^b^	0.86^b^	0.49^c^	1.07^a^	1.04^a^	0.86^c^	0.36^d^
Sn	0.30^a^	0.28^c^	0.29^b^	0.28^c^	0.22^b^	0.21^c^	0.22^b^	0.24^a^
Sr	1.01^c^	1.17^b^	1.27^a^	1.27^a^	0.50^c^	0.47^d^	0.56^b^	0.79^a^
Tl	0.26^b^	0.24^a^	0.28^a^	0.26^b^	0.23^a^	0.23^a^	0.22^b^	0.11^c^
Zn	26.76^a^	24.74^c^	25.86^b^	21.01^d^	23.52^c^	24.88^b^	21.86^d^	27.49^a^
*HREEs*								
Dy	< 0.01	< 0.01	< 0.01	< 0.01	< 0.01	< 0.01	< 0.01	< 0.01
Er	< 0.01	< 0.01	< 0.01	< 0.01	< 0.01	< 0.01	< 0.01	< 0.01
Ho	< 0.01	< 0.01	< 0.01	< 0.01	< 0.01	< 0.01	< 0.01	< 0.01
Lu	< 0.01	< 0.01	< 0.01	< 0.01	< 0.01	< 0.01	< 0.01	< 0.01
Sc	< 0.01	< 0.01	< 0.01	< 0.01	< 0.01	< 0.01	< 0.01	< 0.01
Tb	< 0,01	< 0,01	< 0.01	< 0.01	< 0.01	< 0.01	< 0.01	< 0.01
Tm	0.06^a^	0.05^b^	0.06^a^	0.05^b^	0.04^a^	0.03^b^	0.04^a^	0.04^a^
Y	< 0.01	< 0.01	< 0.01	< 0.01	< 0.01	< 0.01	< 0.01	< 0.01
Yb	< 0.01	< 0.01	< 0.01	< 0.01	< 0.01	< 0.01	< 0.01	< 0.01
*LREEs*								
Ce	0.05^a^	0.04^b^	0.04^b^	0.02^c^	< 0.01	< 0.01	< 0.01	< 0.01
Eu	0.02^a^	0.02^a^	0.02^a^	0.02^a^	< 0.01	< 0.01	< 0.01	0.05
Gd	< 0.01	< 0.01	< 0.01	< 0,01	< 0.01	< 0.01	< 0.01	< 0.01
La	0.05^b^	0.06^a^	0.05^b^	0.05^b^	0.06^a^	0.05^b^	0.04^c^	0.05^b^
Nd	0.41^a^	0.36^b^	0.36^b^	0.30^c^	0.79^a^	0.76^b^	0.65^c^	0.65^c^
Pr	0.27^a^	0.26^b^	0.20^c^	0.21^d^	0.14^a^	0.14^a^	0.12^b^	0.11^c^
Sm	< 0.01	< 0.01	< 0.01	< 0.01	< 0.01	< 0.01	< 0.01	< 0.01

Mean values (n = 3); identical superscripts in a row separately for each species of mushroom denote no significant (α < 0.05) difference between mean values according to Tukey’s HSD test (ANOVA); *HREEs* heavy rare earth elements, *LREEs* light rare earth elements

For the majority of HREEs, their content in fruit bodies of both species was below detection limits, with the exception of Tm, being in the range 0.03–0.06 mg/kg DW. Among LREEs determined in *H. erinaceus*, changes in the content of Ce, La, Nd and Pr with a reduction for Ce, Nd and Pr with regard to different temperatures of drying were recorded. No changes were observed for Eu, while Gd and Sm were below the limit of detection. A reduction of La, Nd and Pr for *L. scabrum* exposed to increased temperature of drying was observed. The other LREEs (Eu, Gd, Nb) were below the limit of detection or no changes were observed.

The obtained results have shown no significant influence of different temperatures of the drying process on content of selected elements in *H. erinaceus* and *L. scabrum* fruiting bodies, which confirms the previous data described by Muyanja et al. (2014). In our opinion, changes in element contents (especially at 70 °C) in mushroom bodies are possible mainly for volatile elements such as the herein studied Sb or Se, as was clearly observed, and also As, not analyzed here. In the case of volatile elements their loss is a common occurrence. Therefore before analysis of the above mentioned elements as well as Hg, a subject of numerous studies of mushrooms, the selection of a suitable drying method or the use of a relatively low temperature is necessary (Hojdová et al. 2015). It is worth noting that a higher drying temperature reduces the time of this stage of sample preparation, although it may be associated with the loss not only of volatile elements but also of heavy metals such as Pb or Zn (Zhang et al. 2001).

Changes in element contents may be more associated with the form of elements than with their total concentration, which confirms the study of Chen et al. (2017a), who described the influence of speciation changes of Cd, Cr and Pb after drying (hot air and freeze drying samples). Mushrooms dried in different ways may be characterized by various contents of numerous elements (Maray et al. 2018), whereas this does not apply to mushrooms dried by the same method but with different temperatures.

## Conclusion

Drying is a process that allows the product to be stored for a longer time and during the off-season, especially for wild growing mushrooms. Drying is also a method of preservation allowing the product to be available all year round. However, temperature significantly affects the content of the analyzed bioactive compounds and antioxidant properties. Losses of TP, phenolic and organic acids and ergosterol content were observed. Changes in elements were probably connected with speciation changes. The obtained results suggested that losses of bioactive compounds probably diminish the pro-health properties of mushrooms.

## Electronic supplementary material

Below is the link to the electronic supplementary material.

Supplementary material 1 (DOCX 14 kb)

Supplementary material 2 (DOCX 18 kb)
