# Supplementary material for: The effect of drying temperature on bioactive compounds and antioxidant activity of Leccinum scabrum (Bull.) Gray and Hericium erinaceus (Bull.) Pers
Source: J Food Sci Technol. 2019 Sep 18;57(2):513–25. doi: 10.1007/s13197-019-04081-1 (PMC7016157; doi:10.1007/s13197-019-04081-1)
Supplement: Supplementary file 1 — Supplementary material 1 (DOCX 14 kb) [file 13197_2019_4081_MOESM1_ESM.docx]

Table S1. Instrumental ICP-OES common parameters – supplementary data

| RF power | 1.2 kW |
| --- | --- |
| Spray chamber | glass cyclonic double pass |
| Nebuliser type | One-Neb (plastic) |
| Nebuliser gas flow | 0.7 L |
| Auxiliary gas flow | 1 L/min |
| Plasma gas flow | 12 L/min |
| CCD temperature | -40°C |
| Polychromator temperature | 35°C |
| Signal accusation | 5 s |
| Repetition | 3 |
| Plasma viewing mode | SVDV* |
| Height of radial plasma observation | 8 mm |

*SVDV – synchronous vertical dual view using dichroic spectral combiner (DSC) technology
