# Supplementary material for: The effect of drying temperature on bioactive compounds and antioxidant activity of Leccinum scabrum (Bull.) Gray and Hericium erinaceus (Bull.) Pers
Source: J Food Sci Technol. 2019 Sep 18;57(2):513–25. doi: 10.1007/s13197-019-04081-1 (PMC7016157; doi:10.1007/s13197-019-04081-1)
Supplement: Supplementary file 2 — Supplementary material 2 (DOCX 18 kb) [file 13197_2019_4081_MOESM2_ESM.docx]

Table S2. Validation data for elemental analysis by ICP-OES

|  | **wavelength** | **DL** | **range** | **uncertainty** |  | **wavelength** | **DL** | **range** | **uncertainty** |
| --- | --- | --- | --- | --- | --- | --- | --- | --- | --- |
|  | nm | mg/kg | mg/kg | **%** |  | nm | mg/kg | mg/kg | **%** |
| **Al** | 394.401 | x | 200-4000 | 6.5 | **Mg** | 285.213 | x | 500-5000 | 4.5 |
| **Al** | 396.152 | 0.0053 | DL-200 | 2.9 | **Mn** | 257.610 | 0.0021 | DL-200 | 1.8 |
| **Ba** | 455.403 | 0.002 | DL-200 | 9.0 | **Na** | 588.995 | 0.26 | DL-1000 | 6.7 |
| **Be** | 313.042 | 0.026 | DL-100 | 12.0 | **Na** | 589.592 | x | 1000-10000 | 3.4 |
| **Ca** | 315.887 | x | 2000-10000 | 6.4 | **Nd** | 406.108 | 0.012 | DL-100 | 6.2 |
| **Ca** | 422.673 | 0.036 | DL-2000 | 2.8 | **Ni** | 231.604 | 0.0092 | DL-200 | 8.0 |
| **Cd** | 214.439 | 0.0026 | DL-200 | 10.1 | **P** | 213.618 | 0.32 | DL-300 | 10.7 |
| **Ce** | 446.021 | 0.023 | DL-100 | 13.6 | **P** | 253.561 | x | 300-4000 | 7.8 |
| **Cr** | 267.716 | 0.0033 | DL-200 | 4.0 | **Pb** | 220.353 | 0.023 | DL-200 | 4.2 |
| **Cu** | 327.395 | 0.0027 | DL-200 | 9.6 | **Pr** | 417.939 | 0.032 | DL-100 | 9.8 |
| **Dy** | 364.540 | 0.023 | DL-100 | 6.8 | **Rb** | 780.026 | 0.045 | DL-100 | 10.1 |
| **Er** | 349.910 | 0.018 | DL-100 | 7.9 | **Sb** | 206.834 | 0.012 | DL-100 | 14.0 |
| **Eu** | 420.504 | 0.034 | DL-100 | 3.0 | **Sc** | 361.383 | 0.024 | DL-100 | 3.0 |
| **Fe** | 238.204 | 0.0084 | DL-1000 | 1.6 | **Se** | 196.026 | 0.011 | DL-100 | 16.4 |
| **Fe** | 261.382 | x | 100-4000 | 0.89 | **Sm** | 442.434 | 0.026 | DL-100 | 9.8 |
| **Gd** | 342.246 | 0.034 | DL-100 | 6.1 | **Sn** | 283.998 | 0.067 | DL-100 | 12.3 |
| **Hg** | 194.164 | 0.023 | DL-100 | 14.6 | **Sr** | 460.733 | 0.0092 | DL-200 | 5.3 |
| **Ho** | 348.484 | 0.031 | DL-100 | 11.9 | **Tb** | 350.914 | 0.027 | DL-100 | 7.3 |
| **Ir** | 205.116 | 0.0097 | DL-100 | 6.5 | **Tl** | 190.794 | 0.024 | DL-200 | 7.3 |
| **K** | 766.491 | 0.34 | DL-1000 | 1.3 | **Tm** | 336.261 | 0.031 | DL-100 | 7.6 |
| **K** | 769.897 | x | 1000-10000 | 0.53 | **Y** | 361.104 | 0.031 | DL-100 | 6.2 |
| **La** | 398.852 | 0.0086 | DL-100 | 6.8 | **Yb** | 328.937 | 0.014 | DL-100 | 3.8 |
| **Lu** | 307.760 | 0.031 | DL-100 | 2.9 | **Zn** | 213.857 | 0.0022 | DL-200 | 3.5 |
| **Mg** | 279.553 | 0.008 | DL-500 | 9.7 |  |  |  |  |  |
